# Annotations

**Published:** 1909-12-18

**Authors:** 


### Annotations.

### The Nobel Prize for Medicine.

All members of the medical profession will cordially congratulate Professor Theodore Kocher, of Berne, who has been awarded the Nobel prize for medicine for this year. The prize, which this year amounts to close upon £8,000, is given annually, in accordance with the terms of Herr Nobel's will, to the man or woman who, in the opinion of the adjudicators, has done most for the advancement of medicine. There can be no question that the award this year will meet with general approval. Professor Kocher's name is known all the world over. His contributions to the advancement of surgical science and art have been numerous and important. As an operator he is second to none, and whoever is venturesome enough to decide the question of the comparative greatness of present-day surgeons will have to take Kocher into serious consideration. His operative work has been pioneering in many departments, and he has proved himself, in others, a close observer and an intrepid follower. We need here only allude to his work on the surgery of the thyroid gland, of ilio-abdominal amputations, of herniæ and of gastric and cranial lesions, in all of which he has laboured in an original and pioneering fashion. Under his *régime* the surgical clinic at Berne has become a Mecca for modern surgeons, and to post-graduate workers he has always proved himself a cordial helper and a kindly and generous teacher. To the man in the street he is scarcely so well known as many of his surgical colleagues; but the profession at large fully recognises his eminence and will join us in tendering him hearty felicitations on the well-deserved honour that has fallen to him through the award of the much coveted prize established by the inventor of dynamite.

### The Late Sir Alfred Jones.

Through the death of Sir Alfred Jones, announced on Tuesday last, the Liverpool School of Tropical Medicine, and indeed medicine generally, loses a staunch and generous supporter. Intimately acquainted with life in tropical countries, acquainted as fully with the economic bearings of such scourges as beri beri, kala azar, sleeping sickness, and yellow fever, and, above all, fully cognisant and appreciative of the good that has been effected by scientific investigation into these problems of tropical disease, Sir Alfred Jones threw himself heart and soul into the establishment and endowment of the Liverpool School, and throughout his lifetime showed himself a fair critic of its work and a fast friend to the workers. He provided the money for the various expeditions organised by the school: it is betraying no secrets when we add that his private purse on more than one occasion supplemented the official funds for special investigations undertaken by students of the school. Such men as he are the men medicine wants as its supporters in this country: men who take a practical interest in the sociological, economic, commercial, and national questions with which medicine in the future will have to deal more closely than it has done in the past: men who do not content themselves with the gift of so many hundreds to found a prize for amateur researchers in some medical school, but who are large visioned enough to take wider views of the necessity for investigation, and who are eager and keen to make themselves personally acquainted with the work that is being done. Such men are few, and we can ill afford to spare them. For that reason we deeply deplore the sudden death of Sir Alfred Jones, one of whose finest memorials, we feel sure, is the success of the school in which he took a founder's pride.

### Preventable Deaths.

A heavy death roll from poisons is recorded in the annual report of the Registrar-General of Births, Deaths, and Marriages which was issued this week. The report covers England and Wales, and deals with the year 1908. The number of cases of death due to accidental poisoning was 270, one-half of which were from scheduled poisons. Of the scheduled poisons, that which proved fatal in the greatest number of cases was opium (and its derivatives), which caused the death of 47 persons; it is satisfactory to note that this is a decrease on the previous year, when no less than 70 cases of accidental death were attributed to opium, laudanum, and morphine. Carbolic acid, taken in mistake for some other substance, produced fatal results in 24 cases, which is nearly double the number recorded in the previous year. Oxalic acid caused eight fatal accidents, chloral hydrate six, and chlorodyne eight. Of the non-scheduled poisons, the one which caused the greatest number of fatal accidents was hydrochloric acid, which caused 13 deaths. The sale of hydrochloric acid is not confined to chemists' shops, but the bottles in which it is sold must be labelled "poison." This regulation, however, did not come into force until April of this year, and it is too early to say what its effect has been on the mortality rate. The sale of ammonia is not even subject to this mild restriction, but nine deaths are attributed to its accidental administration last year. The number of cases in which poisons were taken by "suicides" last year was nearly 600. Naturally the poisons most frequently used were scheduled poisons, these being the most potent. Carbolic acid proved fatal in 98 cases, but oxalic acid was employed in 128 cases of suicide, which is 47 more than in the previous year. Opium and its derivatives were fatal in 60 cases; potassium cyanide caused the death of 35 persons; hydrocyanic acid, 28; and strychnine, 21. Of non-scheduled poisons hydrochloric acid was the most frequent cause of death, proving fatal in 76 cases of suicide, while ammonia was taken with suicidal intent in 11 fatal cases. The number of deaths recorded as due to anæsthetics in 1908 is 209, an increase of 23 on the previous year. Chloroform was the anæsthetic in 98 cases, ether in 16, A.C.E. mixture in 6, chloroform and ether in 4, ether in 2, nitrous oxide in 1, stovaine in 1, and unfortunately in 81 cases the kind is not stated.

